# Supplementary material for: Chromothripsis during telomere crisis is independent of NHEJ, and consistent with a replicative origin
Source: Genome Res. 2019 May;29(5):737–49. doi: 10.1101/gr.240705.118 (PMC6499312; doi:10.1101/gr.240705.118)
Supplement: Supplemental Material [file supp_gr.240705.118_Supplemental_file_1.zip › contigs/annotated_contigs/DB69/contig.2.DB69_length_447_mean_cov_4.02684563758.docx]

**DB69_length_447_mean_cov_4.02684563758**

```
AACCACACCTGACTAATTTTTTATTTTTGTAGAGATGAAGTCTCACTCTAGTGAATTTTCTTATTTTACTTTTTAGAGACAAGGTCTCA
 >chr7:138215641-138215860 + E=3e-120
CTCTGTCACCCAGGCTCGAGTGTAGTGGTATAGCCTTGGCTCACTGCAGCCTCAAACTCCTGGGAAACTGGGACTACAAGCGATCCTCC

TGACTGAGAAGCTGGGACTACAGGGCGTGTGCTGCCA|CTCC|AGTGACTAACCTCCTCCTCTGGCTCCTCCTTGCAGACAGTTATCCC
                                      >chr9:127641048-127641280 - E=4e-128
TTGTTTAGAACACGAATTTCCATTTACCTGGTGGGAACACGAAACAGGAGTCTCTTCTGTTCTGCAAGTTTGATGGGTAAGAGGTAGCC

TTTTTTCAAAGTAGGATTTCCTTTTTCAACTGTTCCAGGAAAGAATCTCTAAGACTGGGTAGCTCACAGCCAGCCAAAGGCAGCTACAT

TTCC
```
